# Biomarker Validation for Aging: Lessons from mtDNA Heteroplasmy Analyses in Early Cancer Detection

**DOI:** 10.4137/bmi.s2253

**Published:** 2009-11-27

**Authors:** Peter E. Barker, Mahadev Murthy

**Affiliations:** 1 Bioassay Methods Group, Biochemical Sciences Division, Bldg 227/B248, NIST, 100 Bureau Drive, Gaithersburg, Maryland; 2 Division of Aging Biology (DAB), National Institute on Aging, 7201 Wisconsin Ave., GW 2C231, Bethesda, MD 20892. Email: peter.barker@nist.gov; mmurthy@mail.nih.gov

**Keywords:** biomarker, aging, cancer, validation, mitochondrial DNA (mtDNA) sequencing, technology, economic impact, healthcare, mitochondriome, mutation, heteroplasmy, early cancer detection, next generation DNA sequencing (NGS), reactive oxygen species (ROS), surface-enhanced laser desorption ionization-based mass spectrometry (SELDI-MS)

## Abstract

The anticipated biological and clinical utility of biomarkers has attracted significant interest recently. Aging and early cancer detection represent areas active in the search for predictive and prognostic biomarkers. While applications differ, overlapping biological features, analytical technologies and specific biomarker analytes bear comparison. Mitochondrial DNA (mtDNA) as a biomarker in both biological models has been evaluated. However, it remains unclear whether mtDNA changes in aging and cancer represent biological relationships that are causal, incidental, or a combination of both. This article focuses on evaluation of mtDNA-based biomarkers, emerging strategies for quantitating mtDNA admixtures, and how current understanding of mtDNA in aging and cancer evolves with introduction of new technologies. Whether for cancer or aging, lessons from mtDNA based biomarker evaluations are several. Biological systems are inherently dynamic and heterogeneous. Detection limits for mtDNA sequencing technologies differ among methods for low-level DNA sequence admixtures in healthy and diseased states. Performance metrics of analytical mtDNA technology should be validated prior to application in heterogeneous biologically-based systems. Critical in evaluating biomarker performance is the ability to distinguish measurement system variance from inherent biological variance, because it is within the latter that background healthy variability as well as high-value, disease-specific information reside.

## Introduction

Biomarker discussions have dominated applied clinical research programs in recent years with the promise of significant clinical utility. Early cancer detection^1^–^3^ and cancer drug development^4^ represent expanding research communities where clinically reliable biomarkers will expedite progress toward improved patient outcomes. Early effective treatment strategies, improved clinical care and reduced healthcare costs represent a few of the diagnostic, screening and prognostic opportunities that may be realized from the availability of rigorously validated biomarkers.

In the drug discovery domain, biomarker validation and qualification discussions have led to the “Fitness-For-Purpose” validation model.^5^ In this, evidence is calibrated against specific applications that would validate and qualify the measurement system and marker for a defined purpose.^6^,^7^ Although applications may differ among aging, cancer, and drug development, the technologies and validation issues overlap. Formal biomarker study design guidelines will continue to evolve rapidly.^8^,^9^

As with prior clinical trial paradigms, the phases of discovery and validation in early cancer detection for screening biomarkers have been recognized.^10^ However, few early detection biomarkers have survived rigorous validation. Similarly in drug development,^11^ the rate of new drug approvals has declined precipitously over the past decade. To address this, the Food and Drug Administration (FDA) is actively seeking ways to expedite and accelerate the path to market for drugs and medical devices.^12^

Despite efforts in many laboratories, disappointingly few candidate biomarkers have been brought to clinical application for early detection of solid tumors. Discovery work narrows the universe of candidate biomarker analytes to those for which data support a link with outcomes in independent specimens. Subsequent application studies are focused on the performance metrics of candidate biomarkers and their measurement technologies for definitive evaluation and classification accuracy in a specified clinical application for a defined clinical purpose. In the final stage of biomarker evaluation, net benefit to patients is determined after utilizing biomarker assay results for clinical intervention.

At biomarker discovery phase, challenges include discovering and quantitating valuable analytes within dynamic and biologically complex matrices such as blood, urine, and sputum. Further challenges include the paucity of biomarker measurement data in healthy populations (normal ranges and sources of variability), and the related issue of appropriate specimen controls. Preanalytical challenges include instability of bioanalytes, RNA and serum proteins for example, and measurements in common archival specimen formats such as formalin-fixed, paraffin embedded (FFPE) tissue blocks. In practice, validation and qualification efforts suffer if the dimensions of the bioanalyte space, normal population variation and bioanalyte stabilities are not addressed fully within the protocols at both the discovery and application phase.

Study design and bias have also been problematic for cancer biomarker validation and qualification. This is illustrated in recent prostate cancer early detection proteomics with surface enhanced laser desorption-ionization based mass spectrometry (SELDI-MS), where biased specimen collection and storage has hampered progress.^13^,^14^ Furthermore, despite significant activity in the private sector on cancer drug development, biomarker discovery efforts have fallen short of anticipated benefits and cost savings.^6^,^15^ With these experiences in mind, the protocols for biomarker validation in specimens from tissue banks and biorepositories are under scrutiny with an eye toward improving technologies, utility and reproducibility among measurements based on specimens from multiple institutions.^16^,^17^ Despite these technical and strategic challenges, early detection of cancer remains in the vanguard of initiatives funded to improve biological measurements and validation of individual biomarkers or biomarker panels.

### Biomarkers of aging

Over the past two decades, the research community targeting aging has also sought predictive and diagnostic biomarkers for physiological aging and age-linked diseases.^18^–^22^ Despite better understanding of the differences between chronological age (measured in years) and physiological age [measured in functional capacity],^22^ progress in finding predictive biomarkers of individual mortality, and in general of physiological aging, has been dismal. Some argue that better definitions of the degenerative processes underlying mortality, not predictors of individual mortality, are the more appropriate goal.^23^

Although there are a few functional human age-related phenotypes on which metrics might be based (for example, loss of muscle function with age, or changes in skin elasticity), there are still many unknowns in human aging phenotypes and how these compare with animal models.^23^–^25^ Unprecedented opportunities set the stage for biomarkers as predictive and diagnostic markers for age-linked conditions, including degenerative processes, with high-throughput technologies and rapid advances in aging.^23^,^26^,^27^ Establishing validated biomarkers would also help in developing targeted interventions for age-linked conditions. Despite significant advances in technology, specific biomarker panels for quantitating physiological age or rate of physiological aging, remain elusive. It is likely that hard-won lessons from biomarker discovery, validation and application studies in early cancer detection, will inform the search for biomarkers of aging as well.

In cancer biomarker work, the goal is to define the precision and reproducibility with which a measured analyte serves a useful clinical function (for example, classification and prediction, surrogate endpoint for a clinical trial, measure of toxic exposure, or as an indicator of best treatment choice).^9^ In parallel, there are experimental clinical interventions that show promise in ameliorating the effects of normal aging independent of disease, for which validated, measurable biomarkers might be useful.

Among the most promising interventions in aging has been dietary caloric restriction (CR). Caloric restriction has reproducible, favorable effects on lifespan and morbidity in a number of metazoan systems.^28^,^29^ However, the magnitude of impact in wild mice is less than inbred laboratory strains.^30^ The molecular aspects of CR have recently been reviewed.^31^,^32^ Dietary supplementation with polyphenolic compounds such as resveratrol may mimic CR at the level of transcriptional profiling in mice.^33^ Specific measureable analytes that serve to quantitate CR and its effect on aging in a reproducible way have not been extensively validated. However, an intriguing recent finding in yeast model systems implies that CR may increase NAD/NADH ratios, in turn upregulating *Sir2* and eliciting a CR-like physiological change.^34^ CR may thus have its impact on longevity through the sirtuins.^35^ It remains to be seen whether this finding will yield a specific quantitative biomarker for aging in higher mammalian and human biology. Recent work^36^ suggests that mammalian SIRT1 (a *Sir2* ortholog) may repress repetitive DNA and genes, and re-localizes to DNA breaks in a manner reminiscent of the yeast system.

Challenges arising from normal individual biological variation and the difficulties in understanding the relative contribution of aging to disease processes have been debated for years by biologists and gerontologists seeking validated biomarkers of physiological aging.^20^ Like cancer biomarkers, biomarker study design for aging, and best practices for evaluation remain undeveloped. Unfortunately, lack of consensus over what constitutes a biomarker of aging (a measureable bioanalyte that assigns biological age) or a predictor of individual mortality, persists. Strategies have been explored for identifying biomarkers of aging in species of long life span.^19^ Despite numerous research publications, the search for aging biomarkers runs parallel to early cancer detection and drug development in that little substantive progress has been reported. While one area of progress may be the technical capability to make sound biological measurements, study design is another area that may substantially improve the situation, especially when study designs appropriate to high-dimensional, highly multiplex data are implemented.^8^

Clearly, in addition to preanalytical processing,^13^ study design issues in pivotal cancer biomarker studies such as overlap between training and test specimen sets, have been problematic.^8^,^9^,^37^,^38^ Although a similar analysis of biomarker study design in aging is not yet available, the high dimensional data resulting from new technologies are increasingly common in studies of experimental aging.^26^,^33^

Since initial searches for aging biomarkers, attention has now turned to high-throughput and rapid, technology-based strategies for revisiting the discovery and validation of biomarkers of aging with new strategies based on aging phenotypes that characterize age-linked functional and degenerative processes. In addition, recent progress in cancer biomarker validation study design^9^ and analysis of design features that compromise results,^8^ might be considered in the evaluation of biomarkers of aging to good effect.

Since the initial search for biomarkers in aging two decades ago,^39^ high-throughput technologies and bioinformatics platforms have vastly improved, as have detailed genomic analyses and databases describing normal human populations.^40^,^41^ The comprehensive quantitation of proteins^42^ and intermediary metabolites^43^,^44^ in complex specimens are additional rapidly evolving biomarker technology areas. Significant standards needs for serum proteomics have been identified.^45^ Like these, comprehensive studies of genomic variability among healthy individuals are few, although several projects aim at normal human sequence variability.^46^ In addition, new high-throughput, cost-effective DNA sequencing platforms, the so-called next generation sequencers (NGS), have emerged recently.^47^ These technologies for deep sequencing promise to revolutionize personalized genomics and medicine by decreasing cost and increasing throughput.

Thus, it is timely given these strides forward to revisit the discovery and validation process that incorporates high-throughput tools for discovering and validating panels of biomarkers, and how these might apply to the development of biomarkers for physiological aging, with special reference to mtDNA.

### Pathways and the systems biology of aging

Comprehensive biological analysis that borrows from the principles of systems engineering has been termed “systems biology”. In this approach to understanding biology, high-dimensional biological measurements are integrated with computational computer models to predict how perturbations in any part of the system will impact the whole. Applications in medicine, drug discovery and engineering have been reviewed.^48^

Aging can be viewed as a system of metabolic or genetic pathways with branch points of importance at many levels of biological organization. The biological basis of cellular senescence and its relationship to organismal aging has been explored.^24^ In physiological aging, a complex network of pathways operate concurrently and independently across the spectrum of biological organization (cell, tissue, and organism), and readily adapt to changing environmental challenges. Despite this complexity, new understanding is beginning to emerge that might reveal network and pathway malfunctions that distinguish normal from age-related pathologies.^49^

For example, the roles of specific pathways in aging have been reported, including the CDKN2a pathway,^50^ the growth hormone/IGF-1 or IIS axis^51^ and the DNA repair-telomere function pathways.^52^ In addition, age as a contributing factor to cellular proliferation and cancer risk is extensively documented in the literature.

For decades, mitochondrial dysfunction and anomalies of oxidative phosphorylation (mutagenetic effects of reactive oxygen species (ROS) production, for example) in aging and cancer have been the focus of numerous studies. Although a mitochondrial theory of aging has emerged recently,^53^–^55^ it remains controversial.^56^–^58^ Mitochondrial DNA point mutations in tumors have been reported.^59^,^60^ Although intriguing, it has been challenging to resolve the role of mtDNA mutations in the biologically complex and intertwined processes of aging and cancer, or the relative clinical value of mtDNA sequence change as a clinical biomarker. In fact, the clinical utility of mtDNA mutation analysis in ovarian cancer has been challenged, based on D-loop sequence and expression levels of six mitochondrial transcripts.^61^ The nature of these possible biological associations remains elusive, however several lines of work have explored mitochondrial changes that might find clinical utility as biomarkers.

The role of epigenetic events such as DNA methylation studies in various nuclear genes in cancer^62^ and aging^63^ has not yielded a consistent story. In part, DNA methylation methods have lacked reproducibility, and steps to address this have been suggested.^64^

Studies of other epigenetic changes such as histone acetylation status, have revealed that the mouse SIRT1 gene (a mammalian ortholog of yeast *Sir2* gene) may be involved in chromatin organization while inhibiting the initiation of DNA replication, and may have a significant role in the biology of longevity and aging.^36^ Further work along these lines may yet yield a molecular biomarker for aging.

Aging biomarkers related to oxidative stress, protein glycation, inflammation, cellular senescence and hormonal dysregulation have been recently reviewed.^22^ To complement this, we therefore focus on mtDNA analysis in human and model systems to illustrate the interactions between technology development and medical application in validation of cellular biomarkers. Two lessons emerge from this review. *First, biological heterogeneity as manifested in healthy development and physiology should be considered prior to analysis of disease states. And second, prior to analysis of biological heterogeneity in either healthy or disease states, the performance metrics and limitations of the analytical technology should be kept in mind.*

### mtDNA analytical technologies

Much has been made of emerging technologies that put within easy reach the detailed human genomic DNA sequence as cells shift from normal to abnormal developmental programs. The key challenge has shifted to data interpretation, from DNA sequence data collection. For mtDNAs, the features most often studied are mtDNA sequence changes (point mutations, deletions, insertions) that differ from the reference mtDNA sequence,^65^,^66^ the degree of sequence heterogeneity in specimens (heteroplasmy), and the total amount of mtDNA present (depletion or amplification), usually on a per-cell basis.

Acute needs for mtDNA analysis include high-throughput, deep sequencing, resolution of mtDNA sequence and variants, amounts and heterogeneity at the single-cell level of resolution. A better understanding of normal mtDNA variability as a function of age, tissue type and nuclear genotype should be developed. To differing degrees, there has been recent technical and scientific progress in each area, although a comprehensive picture of the biology of normal mtDNA and its relationships to disease processes is still emerging.

### DNA copy control in nucleus and mitochondrion

Why are mtDNA copy number determinations important, and how does the biology of copy number control differ when nuclear genes and mitochondrial genes are compared? To evaluate copy number and sequence changes in disease processes, variability in normal human mtDNA is important to establish, especially as reflected in the design of experimental controls.

Most metazoan organisms including human, are represented by haploid and diploid genomic phases that, in terms of genomic copy number, have been considered roughly comparable between genders for autosomal loci. In contrast, mtDNA copy numbers become highly asymmetrical when male and female gametogenesis and early zygote development are compared, with significantly higher and possibly exclusive reliance on maternally derived mtDNA species after fertilization.^67^ In somatic cells, the rule is two copies of nuclear alleles for autosomal loci. However by contrast, mtDNAs are present in hundreds to thousands of mtDNA copies per cell.^68^

Detailed human genomic resequencing has recently uncovered a surprisingly high incidence of nuclear genomic copy number variation (CNV) among phenotypically healthy individuals^41^,^69^ as well as possible links of some forms and degrees of nuclear CNV to diseases of previously unknown etiology.^70^ Similarly, the questions surrounding mtDNA sequence, copy number variation, cellular content control and heteroplasmy in normal and disease processes may prove a productive area of new investigation in medical genetics.

When considering mtDNA metrics as biomarkers for aging, it is appropriate to bear in mind which areas of mtDNA biology remain under active investigation and what facts have been established thus far. With more obscure genetic distribution mechanisms to daughter cells than nuclear chromosomal genes, mitochondria and their genomes are a superb illustration of systems biology interrelatedness at the level of the cell, and represent a highly integrated cellular organelle system with inherent as well as interactive functions. Mitochondria should be viewed both as discrete organelles each containing a genomic complement comprised of many mtDNAs, as well as a subsystem integral to broader cell functioning in critical cellular processes such as bioenergetics^71^ and apoptosis.^72^

Nuclear and mitochondrially-encoded genes both contribute protein components to mitochondrial function, with the vast majority of mitochondrial proteins arising from nuclear genes. Analysis combining mass spectrometry, GFP-tagging, and machine learning, has defined a compendium of 1098 genes and their expression across more than a dozen C57BL/6J strain mouse tissues to define the murine mitochondrial proteome (“mitochondriome”) at an unprecedented level of resolution.^73^ Clearly, with the number of coordinately measured peptides in such approaches, parallel developments in bioinformatics will constitute an important enabling technology for discovery. Such complexity may best be managed by a systems biology framework that incorporates and integrates many types of data bearing on aging.^49^

Compared with nuclear genomic loci that follow Mendelian inheritance, normal mitochondrial gene copy variation represents a less tractable system for whole-animal or somatic cell genetic analysis. In the research laboratory, the availability of mutant ρ^O^ human somatic celll lines^74^,^75^ selected for depletion of mtDNA have made nuclear-mitochondrial substitution experiments possible. Methods for experimentally manipulating metazoan mtDNA have also appeared.^76^ Unlike autosomal nuclear genes that are contributed equally and precisely through parental gametes in mammals, mitochondrial genomes arise primarily from the maternal side at fertilization.^77^–^79^ Beyond the mass excess of maternal mtDNA in oocytes at fertilization, a specific post-fertilization modification of paternal mitochondria (ubiquitination) targets paternal mitochondria for destruction after fertilization in the zygote.^80^ Curiously, paternal mitochondrial targeting may be more active in samespecies matings, than in outcrosses between different, but closely related species.^81^

Although much has been made of the high mutation rates and lack of DNA repair mechanisms within mitochondria in mature mammalian cells and tissues, the early stages of oogenesis appear to have mechanisms for restricting mtDNA genotype.^82^,^83^ Such an oocyte “bottleneck” may function in concert with modification of paternal mitochondria in sperm that are destroyed in the zygote, increasing the likelihood of mtDNA homoplasmy of maternal origin in the newly fertilized ovum. Thus, although conventional nuclear DNA repair systems are not found for mtDNA,^31^ these fertilization-specific processes (sperm mitochondria ubiquitinization and the stochastic oocyte mtDNA “bottleneck”) may accomplish the scanning of mtDNA sequence integrity by alternative means and biological mechanisms that are absent from somatic cells. In animal models, much attention has been devoted to DNA damage and repair in mitochondria,^84^ including generation of transgenic mice with defective excision repair functions associated with the mitochondrial γ-DNA polymerase (POLG),^53^,^57^ a protein that acts in the mitochondrion but which is encoded by a nuclear gene.

At the level of single cells, recent experiments emphasize that intracellular mtDNA populations are not randomly distributed within cells.^85^ The mitochondrial populations resident within a single cell have a coordinate organization based upon the *nucleoid*, an intra-mitochondrial particle consisting of a few defined proteins encoded by nuclear genes (TFAM, mitochondrial single stranded binding protein or mtssBP, DNA polymerase γ [POLG] and *twinkle* DNA helicase) as well as those associated with 2–10 mtDNA molecules that share spatial and temporal functions.^86^,^87^ Recent experiments demonstrate that nucleoids exhibit genetic autonomy from each other within a cell.^87^ Such experiments suggest that mammalian mtDNA nucleoids, with several mtDNAs each, are a type of subcellular, intra-mitochondrial chromosome containing multiple DNA molecules. However, whether the nucleoid is the unit of mtDNA inheritance from parent to daughter cells remains unclear.^82^,^83^

Several independently developing areas of biological research support the idea of biological cross-talk between nuclear and mitochondrial genes. That nucleoid function is genetically and biologically regulated by nuclear genes, is implied by analysis of mtDNA heteroplasmy in centenarians^88^ and the heritability (65%) of mtDNA content in twin studies.^89^ This interaction between mitochondrial and nuclear genes may have implications for understanding how mitochondrial DNA heteroplasmy arises, how it is regulated by the genomic nuclear genes and what its consequences may be for the processes of aging and cancer. A higher rate than in the general population of heteroplasmy has been reported among centenarians and their offspring, and in twin studies.^88^ Thus, variable rates for the development of heteroplasmy among individuals may be an evolutionarily adaptive feature under the control of nuclear genes. Regarding mtDNA sequence variants and longevity, studies in Finnish populations^90^ suggest an association between specific mtDNA sequence variants and longevity, and that the effects may be specific to certain human populations.^91^ More remains to be learned about the variability of mtDNA sequence and content in cells, the biological controlling factors that mediate variability in cells and human populations, and how such variability may relate to health and longevity.

In mice, the presence of specific genetic controls and biases against intraspecific paternal mtDNA is also suggested by paternal mtDNA leakage at the F^1^ generation (related but different species as parents), but not in subsequent backcross generations in intraspecific crosses.^81^ In mice, mitochondrial mutation rates appeared dependent on nuclear genotype in hematopoeitic cells.^92^ These studies also suggest that there remains much more to learn in model organisms about the phenotypes associated with mtDNA sequence heterogeneity, copy number and heteroplasmy in mammalian populations.

In support of the biological idea that longevity may involve mitochondrial-nuclear genome cross-talk, recent experimental work in *Drosophila* in which mitochondrial genotype is varied while controlling for nuclear genotype, implies that mitochondrial genotype has significant effects on longevity of flies, and that the effects may be modulated by the nuclear genetic background.^27^

Thus, before concluding that mtDNA heteroplasmy and mutations in solid tumors are aging-related, disease-specific or normal variability, more work is needed on the normal course of heteroplasmy development and control among aging human individuals and in model animal systems, and its sequence spectrum among healthy tissues. Compared with nuclear genes, control over copy number variants among mtDNAs is not well established in metazoan species. At the very least, the rules of mtDNA copy number constraint are as yet not well understood for mammalian cells. These biological variabilities and uncertainties could compromise claims of mtDNA changes as biomarkers of aging or cancer.

In order to evaluate abnormal mtDNA changes in aging or early cancer, a thorough understanding of normal variability range prior to comparison is critical. The degree to which biological as well as technical variables (measurement and protocol uncertainties) may be confounded, is illustrated by an ongoing debate among experts. These issues are highly relevant to two opposing views of biomarker discovery and validation.

Among some clinicians doing translational work, the molecular identification and details of biomarkers associated with disease and physiological status, may be considered a lower priority than the biomarker’s utility for clinical associations. This point of view held sway in early serum proteomics studies in which a surface-enhanced laser desorption-ionization mass spectrometry (SELDI-MS) pattern was the measured biomarker. In early studies, patterns were validated as disease biomarkers prior to identifying the physical analyte or protein species represented.^93^ In addition, detection algorithms in early studies were subjected to intellectual property (IP) restrictions and nondisclosure. As a result, the explicit experimental methods were published in inadequate detail for independent validation. Since then, the trend has been toward identifying the analytes (identified, proteotypic peptides diagnostic of defined proteins) that can establish disease association with, for example, MALDI-TOF as an improvement over anonymous mass spectra.^94^ There has been discussion of the contentious early analytical issues surrounding cancer biomarker discovery,^8^,^37^,^38^,^95^ and these lessons should inform experimental biomarker qualification/validation strategies in the future in other areas of application, beyond the area of early cancer detection alone.

An opposing point of view held by molecular biologists is that the physical identity of the candidate biomarker analyte (for example, identifying the biomarker protein being measured, not just its anonymous diagnostic mass spectrum trace) is essential to establish improved and sensitive assays, should the initial discovery need a better analytical platform for widespread use. The results of SELDI screening of clinical sera would seem to argue in favor of establishing scientific and biological details on candidate analytes, and most laboratories have adopted peptide identification methods orthogonal to mass spectrometry to address this issue. This further emphasizes the importance of appropriate normal controls and a working knowledge of the dynamic range and concentration limits of normal bioanalyte values. Such lessons should also increase caution in the evaluation of candidate biomarkers based on analytes other than serum proteins.

The confusion and controversy over the physical and chemical identity of biomarkers, have underscored the importance of establishing a molecular basis for biomarkers of all types, but should also bring attention to the previously under appreciated importance of rigorous standard operating procedures (SOPs) for specimen collection, preparation, storage and analysis of biomarker specimens. It is likely that optimization of specimen collection and biobanking will be necessary before the preanalytical variables are better understood, especially as the newest high-resolution, high-dimensional, high-throughput technologies come into clinical research use. Such experiences should inform the path forward as new candidate biomarkers are discovered and evaluated in different areas of application, whether it is early cancer detection, drug development or aging.

### mtDNA variation in health and disease: measurement technology background

The biology of mtDNA sequence, content, distribution and heteroplasmy in mammalian cells (and tissues) is complex, and the factors that control cell content and spectrum of mtDNA are not well understood. In addition, the adequacy of appropriate controls for cancer and aging studies remains under discussion because the background values for mtDNA copy number and sequence heterogeneity are for the most part incomplete, or unknown, in healthy human populations worldwide, and in different tissues within the same individuals.

Given the biological complexity, it is of interest to review technology and platform performance metrics, and the proportion of measurement variance that might be attributed to the analytical tools and platforms used to develop the current picture of mtDNA mutation and heteroplasmy in normal aging and disease. The biology of existing mtDNA sequence and heteroplasmy data in mouse and man is difficult to parse from the point of view of technology, given performance differences in analytical DNA sequencing and quantitation technologies.

### mtDNA sequencing, quantitation technologies and impact

Several levels of mtDNA analysis have contributed to the current understanding of mtDNA variability in human and mammalian model systems. A clear understanding of the biology of mtDNA is important because mtDNA analysis may constitute the basis of critical decisions with significant social impact such as paternity, legal culpability, and identification of human remains.^96^–^98^ The stability of mtDNA over evolutionary time is also the basis for analyses of molecular evolution, and geodistribution of antecedent and contemporary human populations.^99^

The biological variability of human mtDNA is measured against the revised Cambridge reference sequence (rCRS).^66^ Unfortunately, most mtDNA sequence studies in populations focus on sub-regions of the mtDNA genome such as the hypervariable region (incomplete or selective mtDNA sequence analysis), although the trend is toward comprehensive sequencing of the complete mitochondrial genome. Sequence data may also be based on specimens from a variety of different tissue specimens that, in retrospect, may not be biologically equivalent in terms of mtDNA content and variability. Given the fact that there are reports of tissue specificity of mtDNA sequence and heteroplasmy in different anatomical regions of the brain,^100^ tissue of origin of mtDNAs samples, if not properly controlled, may be a confounding factor in attempts to understand the variation of mtDNAs in healthy human populations, and in disease. With regard to lung cancer controls, cigarette smoking in otherwise healthy individuals has been associated with an increase in somatic mtDNA changes in buccal mucosal cells.^101^ Finally, laboratory error confounds published mtDNA data and databases, and is an additional consideration.^102^,^103^ To assay laboratory errors, technical approaches for detecting artifactual mtDNA sequence data have been proposed.^104^

To add to the complexity of mtDNA sequence analysis and its interpretation, the genome also contains several hundred incomplete DNA copies of mtDNA sequence integrated into genomic nuclear DNA in humans and many other species. Such sequences have differing degrees of sequence homology with bona fide mtDNA.^105^,^106^ These nuclear pseudogenes are termed NuMts (nuclear mitochondrial sequences).^107^ In addition to biological uncertainties related to normal tissue and age-specific sequence and content changes, the nuclear mtDNA pseudogenes are a complication with which evaluation of candidate mtDNA biomarker variants must contend.

In model systems such as inbred mice,^108^–^111^ and in human populations,^112^,^113^ significant normal variability in mtDNA sequence is the rule among individuals, strains and populations. Inbred strains of mice that show identity of mtDNA single nucleotide polymorphisms (SNPs) have been catalogued (677 SNPs spanning nucleotides 55 to 16,291) (http://phenome.jax.org).^114^ Recent analysis of inbred mouse strain MRL shows heteroplasmy in tRNA methionine and arginine genes despite a high level of inbreeding.^115^ In human populations, haplogroups consisting of intrapopulational mtDNA sequence features have provided a molecular tool for describing human populations. A series of widely geographically distributed normal individuals has been sequenced recently by chip technology.^113^

In addition to inter-individual mtDNA sequence at a single time point or age, many studies document mtDNA sequence changes with increasing age in healthy inbred mice,^108^,^116^ and in healthy humans.^117^,^118^

Since mtDNA sequence varies normally among healthy individuals in human and murine populations, and within individuals from tissues to tissue, differences detected in association with disease states warrant attention to appropriate controls. For example, whether peripheral blood is the most biologically appropriate control for tissues like lung or bladder tumors might be further investigated.

Despite data that mice^108^,^116^ and humans^88^,^100^,^118^–^122^ show an increase in mtDNA point mutations with age, recent experimental work suggests that the presence of point mutations in murine mtDNA does not significantly lessen life span.^57^ If these data are considered, it would appear that significant gaps still exist in the basic knowledge of normal mtDNA sequence variation, the mutation biology of mtDNA in mammalian species, and the relevance of normal or somatically acquired sequence variants to aging and disease.

#### mtDNA mutations and phenotype

mtDNA sequence changes found in various normal human postmortem^100^ and diseased^59^,^60^ tissues differ from the revised Cambridge reference sequence,^66^ and include point mutations, deletions, insertions, heteroplasmy and mtDNA depletion. Point mutations have been documented as normal variation and form the basis of mtDNA haplogroups in apparently healthy individuals. However, there are also convincing disease associations between mtDNA sequence changes and abnormal phenotypes in native populations and in experimental transgenic mouse systems. In transgenic mice, *twinkle* locus (a nuclear gene encoding mtDNA helicase protein located in the mitochondrion) mutations result in mtDNA deletions and late-onset mitochondrial disease.^123^ mtDNA deletions may be detected in specific human disease states^124^ and in nonmalignant adjacent tissues, as shown in prostate cancer.^125^ Deletions in noncancerous tissues indicate that the utility of such biomarkers for disease diagnostics warrants further investigation.

#### mtDNA and human solid tumors

Early work identified point mutations in colorectal, bladder, head/neck^59^ and primary lung tumors.^60^ In both, the initial work employed Sanger sequencing with radioisotope labels and sequencing gels. The technical accuracy of the mtDNA sequencing results was confirmed and validated independently with dye termination chemistry/capillary sequencing.^126^ It is now clear from these studies that the extent of heteroplasmy and admixture detected by various methods (Sanger sequencing vs. dye-terminator sequencing vs. resequencing chip methods) may have technology-specific performance features, including limits of detection for admixtures. When sequencing technologies differ in limits of detection of heteroplasmy up to an order of magnitude, extreme caution with biological and clinical interpretations of differing levels of DNA admixture is warranted.

Recent analysis of larger patient and healthy populations with MitoChips demonstrates that the extent of normal heteroplasmy and polymorphism in mtDNA has not been fully appreciated.^113^ Among the mutations detected in protein encoding genes, relatively few had obvious biological relevance in that the majority substituted a synonymous codon, leaving the amino acid sequence of the respective gene unchanged. Although scenarios might be envisioned in which mutationally altered tRNAs that functionally insert the identical amino acids during protein synthesis still exert some biological effect, this is another area for future investigation. For example, if an altered mutant mitochondrial tRNA resulted in insertion of the same amino acid, but at a limiting concentration or abnormal rate of incorporation due to the mutation, the mutation might alter the rate of protein synthesis for those proteins rich in that particular amino acid. It is of interest that inbred MRL mice show heteroplasmy of tRNA genes for methionine and arginine as a normal feature, although the biology of this finding is not known.^114^

### Standards and technology for improved mtDNA biomarker utility

The technology of DNA sequencing and mutation detection is rapidly evolving. Methods such as denaturing gradient gel electrophoresis (DGGE)^127^,^128^ are suited to screening experiments and signal the presence of mtDNA heteroplasmy by altered mobility on a gel, but do not detail mtDNA sequence. Appropriate for initial screening, such methods are associated with a stoichiometric limit of quantitation (LOQ) for mtDNA admixtures as low as the 1% level of minor species for some sequence variants, and virtually all heteroplasmy where the minor component is ≥5%.^129^ Such analyses following bands on a denaturing gel alone do not identify the nucleotide bases that are changed. The problem of admixture detection was recognized early in forensic typing of mixed and often degraded DNA samples. For dye terminator sequencing, heteroplasmy can be detected only if the minor species is present at ≥30%.^130^ It has been suggested that methods such as denaturing high performance liquid chromatography (dHPLC) with reported detection levels for admixture at 1%–5%,^113^,^130^–^132^ should be utilized for validation of new heteroplasmy detection methods.

The physical reference mtDNA sequence and its revision^66^ have been established. In addition, physical standards are available for mtDNA analysis such as NIST standard reference materials (SRMs). SRM 2392 and 2392-I are standard reference materials for amplification and sequencing of mtDNAs.^96^,^133^ SRM 2394 is a standard reference material offered with defined levels of mtDNA admixture for human identification and forensics applications.^130^

Intermediate in resolution are the resequencing methods such as MitoChip Versions 1 and 2^113^,^134^–^136^ which report mtDNA sequence changes that match the tiling array features on the chips. These increase throughput and reduce costs for detection of point mutations. Their disadvantage is that they solely detect features that are tiled such as deletions, duplications and insertions, and may not be useful in determining whether mtDNA depletion is present.^113^ In addition, MitoChip resequencing is inefficient in detecting and quantitating mtDNA heteroplasmy.^113^

Thus, DNA sequencing methods characterized by high resolving power for low levels of admixture, as well as high accuracy nucleotide-level sequencing, are of great interest. The most promising are the so-call next generation (NGS) sequencing methods exemplified by the 454 (Roche), Gene Analyzer II (Solexa/Illumina), ABI and Helicos Systems. The power of the 454 system has been demonstrated in recent success with complete sequencing of the degraded mtDNA sequence of 38,000 year old human Neandertal tissues.^99^ The Gene Analyzer II system has shown much technical potential for deep sequencing of nucleosome positions on a genomic scale.^137^ Resolution of mtDNA heteroplasmy at a high level of detail would appear within reach of NGS methods. At present, NGS analysis is approximately five-fold more expensive than chip resequencing.

As DNA sequencing methods move toward lower cost coupled with high throughput, increased resolution and improved limits of quantitation, what is accepted as the normal biological situation for the presence and degree of mtDNA heteroplasmy, may change and evolve with improvements in data. Before disease-related changes in mtDNA sequence can be established, the normal biological background of mtDNA sequence variability must be established.

### Quantitation of mtDNA content: depletion and distribution among cells and single molecule detection

Although not addressing the issue of mtDNA sequence change, measurement of the absolute number of mtDNA molecules in cells is also relevant to biomarker evaluation studies. Ensemble methods based on realtime PCR have been recently reported.^138^,^139^ These, of course, represent averages normalized to nuclear gene targets and may not capture the extent of cell population variability of mtDNA content or mtDNA depletion, although some reports have focused on analysis of singe-cell mtDNA quantitation.^82^,^83^,^118^

Direct visualization of mtDNA nucleoids in cells by microscopy has been possible by detection with DNA dyes, and by *in situ* hybridization. mtDNA methods utilizing DNA intercalating dyes are available, such as DAPI in fixed cells^140^ and PicoGreen in living cells.^141^ DNA hybridization is the basis for another family of mtDNA detection methods. These include *in situ* PCR^142^ and fluorescence *in situ* hybridization.^143^–^145^ Resolution of mtDNA to the level of single nucleotide changes may be achieved through anchored mtDNA mutation detection of single molecules.^146^ Recently, dual-color FISH has made it possible to quantitate different, deleted mtDNAs that functionally trans-complement each other in human cells.^87^ Although not applied to the developmental biology of mtDNA in various tissues with aging as yet, such powerful methods offering single-cell resolution may be another technology-based approach that would add to the understanding of mtDNA variability in healthy aging and disease.

### mtDNA biomarkers and aging: summary and discussion

Better data are needed on the breadth and depth of normal variation in mtDNA sequence and copy number as a function of normal aging and tissue types in mammalian systems. Until normal variation is determined, associations of mtDNA change in somatic cells with diseases will be difficult to evaluate with confidence. Running parallel, the limits of detection (LOD) and limits of quantitation (LOQ) for the technologies should be characterized on healthy specimens prior to disease biomarker studies. Without valid data on the analytical systems established *a priori*, it will be rather difficult to establish and interpret fundamental healthy biological variability. In the long run, it would make more sense and be more cost-effective to work out details through pilot studies prior to planning extensive analytical validations in clinical specimens, or clinical trials.

#### Leveraging parallel efforts in biomarker validation process

Areas that would profit from such technology validation and normal biological studies include early cancer detection. In addition, mtDNA quantity is an important metric in the clinical management of AIDs patients^144^ because some of the drugs used to manage AIDS clinically, inhibit cellular mitochondrial POLG as collateral damage. Thus, better biological studies in normal (healthy) subjects, utilization of new, appropriately validated technologies, and analytical validation of mtDNA content appear to be critical and necessary for pursuing this line of work in areas including healthy aging, early cancer detection and drug development research.

#### Importance of technology evaluations for biomarker measurement: the role of pilot studies

The importance of good analytical validation and physical standards,^147^ and of appropriate study design^38^ prior to embarking on large clinical studies becomes obvious in light of the general lack of success at validating and qualifying biomarkers in aging, drug development and cancer. Another conclusion is that clinical validation of biomarkers must involve multiple study sites to control for local differences in SOPs for specimen collection and storage from cases and controls.

Each of these considerations comes into play in designing better biomarker pilot studies to verify adequate preanalytical processing and technology performance metrics early when studies are of limited scope and cost.

In aging research, useful composite biomarkers or panels might be valuable if one could apply them as predictive or diagnostic markers. Another intended use for such a panel would be to assign biological age, and finally, a further goal might be to predict rate of aging and overall longevity. Obviously, longevity and disease risk are not wholly independent endpoints.

Once the purpose or application of the biomarker measurement is defined, the analytical validation of panels consisting of multiple biomarkers of the same type (multiplex biomarker panel), or panels of biomarkers of various types (DNA mutations or SNP with gene expression data with proteomics, as a composite biomarker panel), is the next step. It is clear there will be few if any single-analyte biomarkers for early cancer detection of solid tumors with the possible exception of specific, rare Mendelian mutations. In drug development work, increasing attention has been paid to the value of biomarkers that are theranostics (therapeutic + diagnostic), or targets in which the druggable target and diagnostic are the same. With the fairly recent realization that single biomarkers may not prove effective, the metrology of how multiple biomarkers can best be applied, is emerging. How such panels will be analytically validated and evaluated to obtain the optimal “fitness-for-use” with a minimum of independent biomarkers or assays, is a novel area of biomarker metrology with which there is limited experience at present. In parallel with novel candidate biomarkers emerging in many areas such as microRNA diagnostics,^148^ the best systematic approach to analytical validation and qualification of biomarkers and biomarker panels in clinical trials is currently undergoing rapid development. In any case, it will be useful to capture the prior experience of the biomarker validation community in academic, government and private sector applications as this field moves forward in many diverse applications.

## Figures and Tables

**Table 1.** Challenges to evaluation of mtDNA-based disease biomarkers.

**Biological Heterogeneity/mtDnA sequence** Normal mtDNA sequence polymorphisms between human populations (haplogroups) mtDNA sequence change vs. developmental age, within populations mtDNA sequence change vs. tissue type, within individuals mtDNA sequence change in disease vs. normal cells Bona fide mtDNA sequences vs. nuclear mtDNAs pseudogenes (NuMTs) Deleted mtDNA sequences, normal vs. disease
**Biological Heterogeneity/mtDnA content** mtDNA number content among cells, tissues, individuals and populations mtDNA number content, normal vs. disease vs. therapies
**Measurement Issues for evaluation of mtDnA as a Biomarker** Comprehensive sequence vs. subgenomic sequence The true limit of detection (LOD) for sequencing technologies The technology LOD for mtDNA sequence admixtures Extent of mtDNA reference databases corresponding to normal mtDNA values
